# Acute psychological impact on COVID-19 patients in Hubei: a multicenter observational study

**DOI:** 10.1038/s41398-021-01259-0

**Published:** 2021-02-18

**Authors:** Minghuan Wang, Caihong Hu, Qian Zhao, Renjie Feng, Qing Wang, Hongbin Cai, Zhenli Guo, Kang Xu, Wenjing Luo, Canshou Guo, Sheng Zhang, Chunfa Chen, Chunli Zhu, Hongmin Wang, Yu Chen, Li Ma, Peiyan Zhan, Jie Cao, Shanshan Huang, Mia Jiming Yang, Yuxin Fang, Suiqiang Zhu, Yuan Yang

**Affiliations:** 1 Department of Neurology and Psychiatry, Tongji Hospital, Tongji Medical College, Huazhong University of Science and Technology, Wuhan, 430030 China; 2 Wuhan No. 9 Hospital, Wuhan, 430030 China; 3 Hubei Provincial Hospital of Integrated Chinese and Western Medicine, Wuhan, 430015 China; 4 General Hospital of the Yangtze River Shipping, Wuhan Brain Hospital, Wuhan, 430000 China; 5 People’s Liberation Army General Hospital of Central Theatre Command, Wuhan, 430000 China; 6 Jianghan University Hospital, Wuhan, 430015 China; 7 Liyuan Hospital, Tongji Medical College, Huazhong University of Science and Technology, Wuhan, 430077 China; 8 The Second Hospital of Huangshi, Hubei, 430000 China; 9 Wuhan Red Cross hospital, Wuhan, 430015 China; 10 The Huanggang Central Hospital, Wuhan, 438000 China; 11 The Third People’s Hospital of Hubei Province, Wuhan, 430022 China; 12 Jingzhou Central Hospital, Second Clinical Medical College of Yangtze University, Hubei, 430000 China; 13 The Central Hospital of Wuhan, Wuhan, 430014 China; 14 Institute for Healthcare Management and Health Science, Faculty of Law, Business & Economics, University of Bayreuth, Bayreuth, Germany; 15 Wuhan Britain-China School, Wuhan, 430030 China

**Keywords:** Human behaviour, Depression

## Abstract

We conducted a multicentre cross-sectional survey of COVID-19 patients to evaluate the acute psychological impact on the patients with coronavirus disease 2019 (COVID-19) during isolation treatment based on online questionnaires from 2 February to 5 March 2020. A total of 460 COVID-19 patients from 13 medical centers in Hubei province were investigated for their mental health status using online questionnaires (including Patient Health Questionnaire-9, Generalized Anxiety Disorder-7, Patient Health Questionnaire-15, and Insomnia Severity Index scales). Among all 460 COVID-19 patients, 187 (40.65%) of them were healthcare workers (HCWs). 297 (64.57%) of them were females. The most common psychological problems were somatization symptoms (66.09%, *n* = 304), followed by depression (53.48%, *n* = 246), anxiety (46.30%, *n* = 213), problems of insomnia (42.01%, *n* = 171), and then self-mutilating or suicidal thoughts (23.26%, *n* = 107). Of all the patients, 15.65% (*n* = 72) had severe somatization symptoms, and 2.83% (*n* = 13) had severe (almost every day) self-mutilating or suicidal thoughts. The most common psychological problems for HCWs were somatization symptoms (67.84%, *n* = 125), followed by depression (51.87%, *n* = 97), anxiety (44.92%, *n* = 84), problems of insomnia (36.18%, *n* = 55), and then self-mutilating or suicidal thoughts (20.86%, *n* = 39). Patients with lower education levels were found to be associated with higher incidence of self-mutilating or suicidal thoughts (odds ratio [OR], 2.68, 95% confidence interval [95% CI], 1.66–4.33 [*P* < 0.001]). Patients with abnormal body temperature were found to be associated with higher incidence of self-mutilating or suicidal thoughts (OR, 3.97, 95% CI, 2.07–7.63 [*P* < 0.001]), somatic symptoms (OR, 2.06, 95% CI, 1.20–3.55 [*P* = 0.009]) and insomnia (OR, 1.66, 95% CI, 1.04–2.65 [*P* = 0.033]). Those with suspected infected family members displayed a higher prevalence of anxiety than those without infected family members (OR, 1.61, 95% CI, 1.1–2.37 [*P* = 0.015]). Patients at the age of 18–44 years old had fewer somatic symptoms than those aged over 45 years old (OR, 1.91, 95% CI, 1.3–2.81 [*P* = 0.001]). In conclusion, COVID-19 patients tended to have a high prevalence of adverse psychological events. Early identification and intervention should be conducted to avoid extreme events such as self-mutilating or suicidal impulsivity for COVID-19 patients, especially for those with low education levels and females who have undergone divorce or bereavement.

## Introduction

The outbreak of coronavirus disease 2019 (COVID-19) first emerged in Wuhan, Hubei Province, China, in December 2019^1–4^. The pandemic of COVID-19 has led to the declaration of Public Health Emergency of International Concern (PHEIC) by the World Health Organization (WHO) on 30 January 2020^5^. To fight against this emergent infectious disease, drastic measures have been taken, such as closing schools and canceling sporting events and other gatherings^6^. Many big cities like Wuhan were forced to undergo quarantine to control the transmission of this infectious disease. To make things worse, very few treatments had been proved effective for this disease until recently^7–9^. People in the swirl of this catastrophic epidemic would inevitably develop varying degrees of anxiety, depression, panic, and insomnia^10,11^. Healthcare workers (HCWs) were at high risk of infecting COVID-19 owing to insufficient medical supplies at the early stage of the epidemic^12,13^. It was reported that the infected HCWs accounted for 29% of all hospitalized COVID-19 patients at the beginning of the epidemic^14^. A multi-national and -center study found that the prevalence of physical symptoms was significantly associated with the adverse psychological outcomes of depression, anxiety, stress, and post-traumatic stress disorder (PTSD) among the HCWs, who were involved in caring for the COVID-19 patients in India and Singapore during the initial stages of COVID-19 pandemic.^13^ Another Singapore study suggested that the nonmedical HCWs were found under even higher risk in anxiety, stress, and subjective distress caused by traumatic events during the outbreak of the pandemic^15^.

Studies on the psychological characteristics of quarantined Severe Acute Respiratory Syndrome (SARS) patients revealed that different levels of anxiety, depression, insomnia, and other psychological stress reactions occurred during the SARS outbreak^16,17^. Quarantined COVID-19 patients including infected HCWs might be facing potential social isolation^18–20^. Moreover, people became surrounded by negative information and rampant misinformation, which had inevitably exaggerated people’s fear, panic, as well as distress. In such a situation, a range of psychological health problems can be anticipated but have yet to be evaluated^21^. Therefore, the purpose of this study is to assess the mental health of COVID-19 patients through an online questionnaire and provide a basis for future psychological intervention.

## Methods

### Study des**i**gn and participants

This study was a multicenter cross-sectional study. A total of 460 COVID-19 patients from 13 medical centers in Hubei Province participated in this study, covering ~5% of the total hospitalized cases in Hubei province at that time. We used a stratified random sampling method to obtain a representative sample, which proportionated to the number of patients admitted to this hospital. We stratified patients in HCWs and others within each selected hospital and, after that, randomly selected them from each center. We included a substantial number of HCWs in this survey aimed to study the psychological problems of HCWs. The sample size in each hospital was proportionated to the number of patients admitted in this hospital, and at least 30% of them were HCWs. The severity of COVID-19 was determined based on the WHO Interim Guidelines document^22^. We only included the COVID-19 patients who were not in critical conditions in our survey. The health conditions of patients were evaluated by the physicians of the isolation wards. Only those who were capable of completing the survey were enrolled in the survey.

Data were collected through anonymous online questionnaires using PHQ-9 (Patient Health Questionnaire-9), GAD-7 (Generalized Anxiety Disorder-7), PHQ-15 (Patient Health Questionnaire-15), and ISI (Insomnia Severity Index) scales. Sociodemographic information was also collected through anonymous online questionnaires. The senior investigators performed quality control by checking the collected questionnaires daily. Informed consent was obtained from all subjects and the study was approved by the institutional ethics board of Tongji Hospital, Tongji Medical College of Huazhong University of Science and Technology (ID: TJ-IRB20200101).

### Measures

PHQ-9 Scale was used to measure the depression symptoms^23,24^. A cutoff of ≥5 has been recommended for considering depression. PHQ-9 scores of 5, 10, 15, and 20 represented mild, moderate, moderately severe, and severe depression, respectively. GAD-7 Scale was used to identify anxiety disorders.^25^ A cutoff score of ≥5 is recommended for considering clinically important anxiety symptoms, which provides adequate sensitivity (82.0%) and specificity (77.0%). GAD-7 scores of 5, 10, and 15 represented mild, moderate, and severe anxiety disorders, respectively. PHQ-15 Scale was used to measure the somatic symptoms severity^26^. A cutoff of ≥5 has been recommended for considering somatization symptoms, which provides adequate sensitivity (88.0 percent) and specificity (88.0 percent). PHQ-15 scores of 5, 10, and 15 represented mild, moderate, and severe somatic symptoms, respectively. ISI Scale was used to measure the severity of insomnia^27^. ISI scores of 8, 15, and 22, represented mild, moderate, and severe insomnia, respectively, and previously used during the past COVID-19 research^28,29^. A cutoff of ≥10 has been recommended for detecting insomnia, which provides adequate specificity (87.7%) and sensitivity (86.1%). Self-mutilating or suicidal thoughts were acquired from the last item of PHQ-9 Scale as “Thoughts that you would be better off dead or of hurting yourself in some way”^30^.

### Statistical analysis

Date was generated from the online survey system. Descriptive statistics of categorical data were expressed by a number of cases and percentage. Multiple logistic regression models were used to explore the risk factors related to psychological problems in COVID-19 patients and HCWs with COVID-19, respectively. This study was a multicentre design. Therefore, the mixed effect model was selected to analyze the data. Considering that the survey data of different research objects in the same medical institution might be aggregated, when building the model, the medical institution was set as a random effect. SPSS19.0 was subsequently used for statistical analysis. *P* value ≤0.05 was defined as the standard significance level.

## Results

**Fig. 1:**
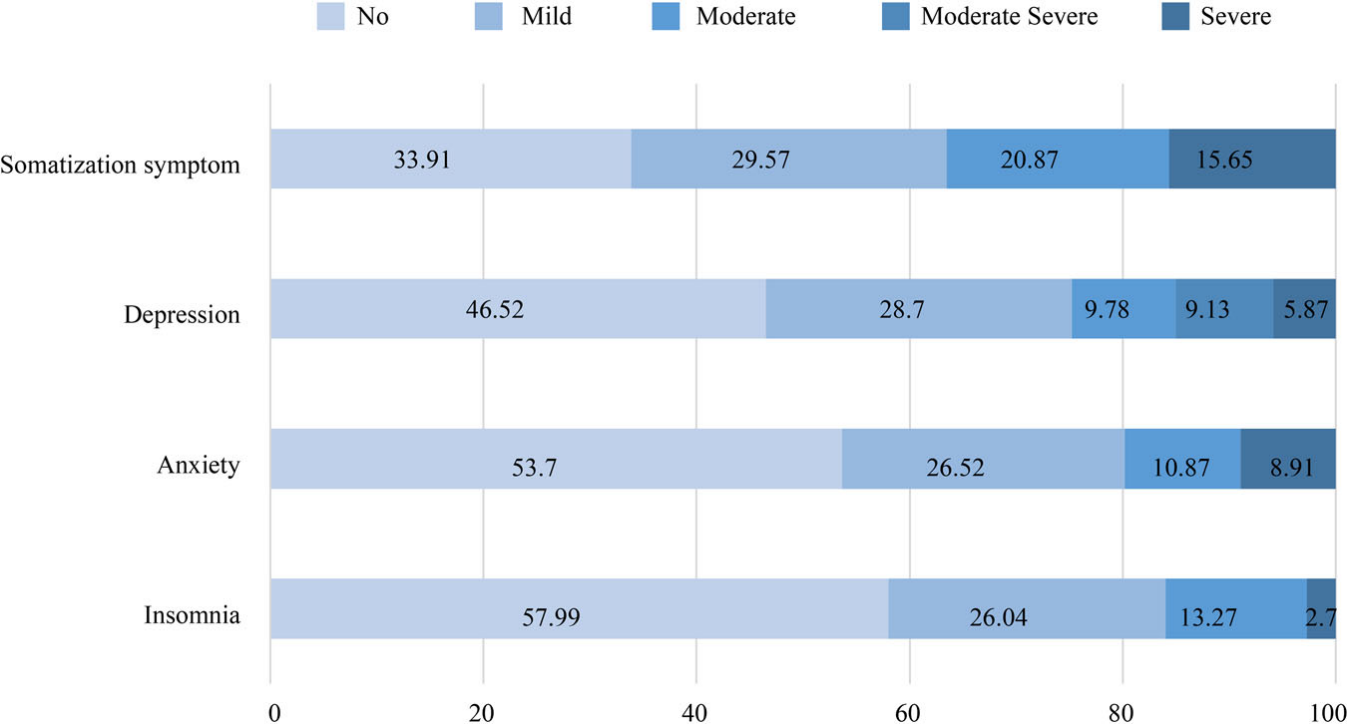
Distribution and severity of various psychological problems in COVID-19 patients. *Y* axis is the names of each kind of mental problems. *X* axis shows the percentage.

A total of 460 COVID-19 patients from 13 medical centers in Hubei provinces were included in our final survey, with a response rate of 92.3% (460/498). Among all COVID-19 patients, 187 (40.65%) of them were HCWs and 297 (64.57%) were females. Most individuals were in the age intervals of 18–44 years old (222 [48.26%]), and 79 (17.17%) were adolescents. In all, 84 (18.26%) patients were living alone. Nearly half of the patients (42.17%) had family members who were infected. Other characteristics of the survey population are shown in Table 1. The distribution of psychological problems and the severity are displayed in Fig. 1. The most common psychological problems were somatization symptoms (66.09%, *n* = 304), followed by depression (53.48%, *n* = 246), anxiety (46.30%, *n* = 213), problems of insomnia (42.01%, *n* = 171), and then self-mutilating or suicidal thoughts (23.26%, *n* = 107). Of all, 15.65% (*n* = 72) patients had severe somatization symptoms; 8.91% patients (*n* = 41) had severe anxiety; 5.87% (*n* = 27) patients had severe depression; 2.83% (*n* = 13) patients had severe self-mutilating or suicidal thoughts; 2.70% (*n* = 11) patients had severe problems of insomnia. The most common psychological problems for HCWs were somatization symptoms (67.84%, *n* = 125), followed by depression (51.87%, *n* = 97), anxiety (44.92%, *n* = 84), problems of insomnia (36.18%, *n* = 55), and then self-mutilating or suicidal thoughts (20.86%, *n* = 39).

**Table 1:** Sociodemographic characteristics of surveyed COVID-19 patients.

Characteristics	All respondents		HCWs	
	*N*	Percentage (%)	*N*	Percentage (%)
*Gender*				
Male	163	35.43	38	20.32
Female	297	64.57	149	79.68
*Age*, *year*				
≤17	79	17.17	32	17.11
18~44	222	48.26	129	68.98
≥45	159	34.57	26	13.91
*Education level*				
Senior high school or below	161	35	26	13.9
Above Senior high school	299	65	161	86.1
*Marital status*				
Unmarried	100	21.74	61	32.62
Married	319	69.35	118	63.1
Divorce or windowed	41	8.91	8	4.28
*Dwelling state*				
Live alone	84	18.26	47	25.13
Live together	376	81.74	140	74.87
*Concomitant disease*				
No	325	70.65	158	84.49
Yes	135	29.35	29	15.51
*Nucleic acid test*				
Positive	175	38.04	57	30.48
Negative	285	61.96	130	69.52
*Fever*				
Yes	411	89.35	171	91.44
No	49	10.65	16	8.56
*Need oxygen inhalation*				
No	339	73.7	150	80.21
Yes	121	26.3	37	19.79
*Family members’ infection*				
Confirmed infection	134	29.13	30	16.04
Suspected	60	13.04	22	11.77
No infection	266	57.83	135	72.19
*Psychological counseling*				
No	352	76.52	141	75.4
Yes	108	23.48	46	24.6
*Suicidal ideation*				
No	353	76.74	148	79.14
Yes	107	23.26	39	20.86
*Depression*				
No	214	46.52	90	48.13
Yes	246	53.48	97	51.87
*Anxiety*				
No	247	53.7	103	55.08
Yes	213	46.3	84	44.92
*Somatization symptoms*				
No	156	33.91	62	33.16
Yes	304	66.09	125	66.84
*Stress response*				
No	350	86	133	87.5
Yes	57	14	19	12.5
*Insomnia*				
No	236	57.99	97	63.82
Yes	171	42.01	55	36.18

*HCWs* healthcare works.

We did the multiple logistic regression analysis to explore the risk factors related to psychological problems in COVID-19 patients. (Table 2 and Supplementary Table S1–S6) Female COVID-19 patient individuals reported a higher incidence of in somatization symptoms (odds ratio [OR], 2.54; 95% confidence interval [95% CI], 1.77–3.63 [*P* < 0.001]), insomnia (OR, 1.49; 95% CI, 1.07–2.09 [*P* = 0.019]), anxiety (OR, 1.64; 95% CI, 1.21–2.23 [*P* = 0.001]), suicidal ideation (OR, 1.97; 95% CI, 1.17–3.32 [*P* = 0.011]), stress response (OR, 3.67; 95% CI, 1.65–8.15 [*P* = 0.001]), and depression (OR, 2.17; 95% CI, 1.48–3.18 [*P* < 0.001]) than male patients. Those who had undergone divorce or bereavement reported higher rate of somatization symptoms (OR, 2.87; 95% CI, 1.49–5.52 [*P* = 0.002]), insomnia (OR, 2.02; 95% CI, 1.10–3.71 [*P* = 0.023]), anxiety (OR, 2.97; 95% CI, 1.70–5.20 [*P* < 0.001]), suicidal ideation (OR, 3.71; 95% CI, 1.52–9.01 [*P* = 0.004]), stress response (OR, 3.94; 95% CI, 1.33–11.67 [*P* = 0.013]), and depression (OR, 3.60; 95% CI, 1.79–7.25 [*P* < 0.001]) than those who were unmarried. Those who were married had more somatic symptoms (OR, 1.67; 95% CI, 1.11–2.52 [*P* = 0.014]), anxiety (OR, 2.26; 95% CI, 1.49–3.43 [*P* < 0.001]), and depression (OR, 1.95; 95% CI, 1.24–3.07 [*P* = 0.004]) compared with those single individuals. Patients with lower education levels tended to have higher incidence of self-mutilating or suicidal thoughts (OR, 2.68; 95% CI, 1.66–4.33 [*P* < 0.001]) and lower stress response (OR, 0.51; 95% CI, 0.26–1.00 [*P* = 0.049]). Notably, we found that, over all, patients who had fever tend to have a higher incidence of self-mutilating or suicidal thoughts (OR, 3.97; 95% CI, 2.07–7.63 [*P* < 0.001]), somatic symptoms (OR, 2.06; 95% CI, 1.20–3.55 [*P* = 0.009]), and insomnia (OR, 1.66; 95% CI, 1.04–2.65 [*P* = 0.033]). Those who had family members suspected as infected displayed a much higher level of anxiety than those without infected family members (OR, 1.61; 95% CI, 1.10–2.37 [*P* = 0.015]). In addition, patients at the age of 18–44 had more somatic symptoms in comparison with the patients >45 years old (OR, 1.91; 95% CI, 1.30–2.81 [*P* = 0.001]). And patients no >17 years old had less insomnia in comparison with the patients >45 years old (OR, 0.42; 95% CI, 0.25–0.70 [*P* = 0.001]) (Table 2).

**Table 2:** Factors associated with psychological problems in COVID-19 patients.

	Somatization symptoms		Insomnia		Anxiety		Suicidal ideation		Stress response		Depression	
	OR (95% CI)	*P* value	OR (95% CI)	*P* value	OR (95% CI)	*P* value	OR (95% CI)	*P* value	OR (95% CI)	*P* value	OR (95% CI)	*P* value
*Gender*												
Female	2.54 (1.77–3.63)	<0.001	1.49 (1.07–2.09)	0.019	1.64 (1.21–2.23)	0.001	1.97 (1.17–3.32)	0.011	3.67 (1.65–8.15)	0.001	2.17 (1.48–3.18)	<0.001
Male	1		1		1		1		1		1	
*Marital status*												
Divorce/bereavement	2.87 (1.49–5.52)	0.002	2.02 (1.10–3.71)	0.023	2.97 (1.70–5.20)	<0.001	3.71 (1.52–9.01)	0.004	3.94 (1.33–11.67)	0.013	3.60 (1.79–7.25)	<0.001
Married	1.67 (1.11–2.52)	0.014	1.29 (0.82–2.03)	0.275	2.26 (1.49–3.43)	<0.001	1.72 (0.91–3.28)	0.096	1.54 (0.70–3.37)	0.279	1.95 (1.24–3.07)	0.004
Unmarried	1		1		1		1		1		1	
*Psychological counseling*												
Yes	1.60 (1.10–2.33)	0.013	1.73 (1.20–2.49)	0.003	1.87 (1.38–2.52)	<0.001	1.81 (1.07–3.05)	0.026	4.64 (2.40–9.01)	<0.001	1.74 (1.15–2.62)	0.008
No	1		1		1		1		1		1	
*Need oxygen inhalation*												
Yes	2.61 (1.73–3.94)	<0.001	1.50 (1.04–2.15)	0.029	1.69 (1.25–2.27)	0.001	_	_	_	_	2.60 (1.74–3.87)	<0.001
No	1		1		1		_	_	_	_	1	
*Fever*												
Yes	2.06 (1.20–3.55)	0.009	1.66 (1.04–2.65)	0.033	_	_	3.97 (2.07–7.63)	<0.001	_	_	_	_
No	1		1		_	_	1		_	_	_	_
*Education level*												
Senior high school or below	_	_	_	_	_	_	2.68 (1.66–4.33)	<0.001	0.51 (0.26–1.00)	0.049	_	_
Above senior high	_	_	_	_	_	_	1		1		_	_
*Age*, *year*												
≤17	1.26 (0.77–2.04)	0.356	0.42 (0.25–0.70)	0.001	_	_	_	_	_	_	_	_
18–44	1.91 (1.30–2.81)	0.001	0.71 (0.50–1.00)	0.051	_	_	_	_	_	_	_	_
≥45	1		1		_	_	_	_	_	_	_	_
*Family members’ infection*												
Confirmed infection	_	_	_	_	1.15 (0.84–1.58)	0.381	_	_	_	_	_	_
Suspected	_	_	_	_	1.61 (1.10–2.37)	0.015	_	_	_	_	_	_
No infection	_	_	_	_	1		_	_	_	_	_	_
*Concomitant disease*												
Yes	1.82 (1.26–2.62)	0.002	_	_	_	_	_	_	_	_	_	_
No	1		_	_	_	_	_	_	_	_	_	_

The result of the multiple logistic regression analysis of HCWs is presented in Table 3. Female HCWs also reported a higher incidence of somatization symptoms (OR, 2.46; 95% CI, 1.09–5.59 [*P* < 0.001]) than males. Lower education levels tended to have more suicidal ideation (OR, 4.81; 95% CI, 1.41–16.43 [*P* < 0.001]). Those who were unmarried reported a lower rate of suicidal ideation (OR, 0.05; 95% CI, 0.01–0.40 [*P* = 0.005]), insomnia (OR, 0.03; 95% CI, 0.001–0.48 [*P* = 0.014]), and anxiety (OR, 0.06; 95% CI, 0.01–0.64 [*P* = 0.020]) than those who had undergone divorce or bereavement. And those who were married had less suicidal ideation (OR, 0.09; 95% CI, 0.02–0.59 [*P* = 0.012]), and insomnia (OR, 0.05; 95% CI, 0.003–0.68 [*P* = 0.025]) than those who had undergone divorce or bereavement. The HCWs who required oxygen inhalations had more anxiety (OR,10.20; 95% CI, 3.10–33.33 [*P* < 0.001]), somatization symptoms (OR, 8.2; 95% CI, 1.65–40.00 [*P* < 0.010]), insomnia (OR,16.95; 95% CI, 3.8–90.91 [*P* = 0.001]), and depression (OR, 5.41; 95% CI, 1.79–16.39 [*P* < 0.001]) than the others. Those who need psychological counseling reported a higher incidence of somatization symptoms (OR, 3.44; 95% CI, 1.26–9.35 [*P* = 0.016]), insomnia (OR, 5.44; 95% CI, 1.66–17.86 [*P* = 0.006]), and anxiety (OR, 1.33; 95% CI, 1.09–6.33 [*P* = 0.032]).

**Table 3:** Factors associated with psychological problems in COVID-19 HCWs.

	Somatization symptoms		Insomnia		Anxiety		Suicidal ideation		Stress response		Depression	
	OR (95% CI)	*P* value	OR (95% CI)	*P* value	OR (95% CI)	*P* value	OR (95% CI)	*P* value	OR (95% CI)	*P* value	OR (95% CI)	*P* value
*Gender*												
Female	2.46 (1.09–5.59)	0.031	1.19 (0.47–3.03)	0.709	1.14 (0.49–2.65)	0.763	1.08 (0.39–2.96)	0.881	0.98 (0.28–3.47)	0.971	1.54 (0.71–3.33)	0.275
Male	1		1		1		1		1		1	
*Age*, *year*												
<35	1.76 (0.56–5.54)	0.330	3.12 (0.72–13.50)	0.127	2.37 (0.72–7.84)	0.157	0.96 (0.27–3.49)	0.954	4.86 (0.35–68.38)	0.240	2.72 (0.90–8.21)	0.076
35–45	1.63 (0.48–5.52)	0.431	1.70 (0.38–7.55)	0.483	1.11 (0.31–3.93)	0.871	0.57 (0.14–2.38)	0.438	3.53 (0.25–50.10)	0.348	1.31 (0.41–4.13)	0.645
>45	1		1		1		1		1		1	
*Education level*												
Senior high school or below	0.30 (0.09–1.00)	0.049	0.64 (0.14–2.89)	0.556	0.39 (0.09–1.60)	0.187	4.81 (1.41–16.43)	0.013	0.073 (0.01–0.93)	0.044	0.48 (0.15–1.51)	0.210
Above senior high	1		1		1		1		1		1	
*Marital status*												
Unmarried	0.21 (0.02–2.64)	0.228	0.03 (0.001–0.48)	0.014	0.06 (0.01–0.64)	0.020	0.05 (0.01–0.40)	0.005	0.80 (0.03–21.38)	0.890	0.16 (0.02–1.26)	0.082
Married	0.22 (0.02–2.46)	0.219	0.05 (0.003–0.68)	0.025	0.23 (0.03–1.90)	0.171	0.09 (0.02–0.59)	0.012	0.86 (0.05–16.20)	0.921	0.30 (0.05–2.01)	0.214
Divorce/bereavement	1		1		1		1		1		1	
Dwelling state												
Live alone	0.73 (0.28–1.90)	0.512	1.01 (0.34–3.00)	0.984	0.67 (0.24–1.82)	0.427	0.80 (0.25–2.54)	0.703	2.97 (0.79–11.11)	0.105	0.68 (0.29–1.64)	0.394
Live together	1		1		1		1		1		1	
*Concomitant disease*												
No	1.56 (0.54–4.53)	0.410	2.84 (0.77–10.46)	0.116	3.11 (0.95–10.24)	0.061	2.10 (0.57–7.72)	0.265	0.74 (0.15–3.65)	0.704	1.19 (0.45–3.14)	0.727
Yes	1		1		1		1		1		1	
*Nucleic acid test*												
Nucleic acid positive	1.38 (0.62–3.07)	0.43	1.37 (0.57–3.32)	0.479	0.91 (0.41–2.01)	0.807	0.65 (0.25–1.66)	0.363	0.80 (0.23–2.78)	0.721	1.425 (0.69–2.92)	0.345
Nucleic acid negative	1		1		1		1		1		1	
*Fever*												
Yes	2.59 (0.20–33.33)	0.463	0.20 (0.02–1.76)	0.144	1.06 (0.19–6.02)	0.944	0.97 (0.19–4.90)	0.973	0.86 (0.07–10.99)	0.909	1.15 (0.23–5.88)	0.866
No	1		1		1		1		1		1	
*Need oxygen inhalation*												
Yes	8.20 (1.65–40.00)	0.010	16.95 (3.18–90.91)	0.001	10.20 (3.10–33.33)	<0.001	2.44 (0.83–7.14)	0.104	4.33 (0.91–20.41)	0.065	5.41 (1.79–16.39)	0.003
No	1		1		1		1		1		1	
*Family members’ infection*												
Confirmed infection	1.01 (0.33–3.10)	0.986	0.48 (0.14–1.72)	0.257	2.21 (0.74–6.58)	0.154	1.22 (0.40–3.73)	0.730	2.66 (0.65–10.95)	0.173	1.11 (0.41–3.01)	0.839
Suspected	0.68 (0.22–2.15)	0.515	1.82 (0.50–6.63)	0.363	3.03 (0.99–9.28)	0.053	0.99 (0.26–3.60)	0.982	0.44 (0.04–4.47)	0.482	1.50 (0.52–4.31)	0.455
No infection	1		1		1		1		1		1	
*Psychological counseling*												
Yes	3.44 (1.26–9.35)	0.016	5.44 (1.66–17.86)	0.006	2.63 (1.09–6.33)	0.032	1.33 (0.52–3.39)	0.552	2.29 (0.63–8.33)	0.206	1.88 (0.85–4.18)	0.118
No	1		1		1		1		1		1	

## Discussion

The COVID-19 pandemic is now a global health crisis and societal emergency^31,32^. A rapid escalation of COVID-19 cases and deaths had been reported in the world^33–35^. Until now, >72 million people had been infected. The appearance and continuation of these dire situations may lead to serial psychological problems in society, especially for patients who were isolated for infection. Our study demonstrated that the incidence rate of depression, anxiety, sleeping disorders, and physical disorders of COVID-19 patients was 49.05%, 56.60%, 67.92%, 69.80%, respectively, all of which were significantly higher than those of the general population. Comparing with a longitudinal study, the prevalence of anxiety and depression in the general population accounted for 28.8% and 16.5%, respectively. Although the levels of stress, anxiety, and depression have remained stable in the face of the explosion of infection cases and no clinical evidence of the reduction in the psychological impact on the general population either^36^. Another study revealed the worst situation among the psychiatric patients, the incidence of physical symptoms in patients with mental illness was 30.3%, and the negative psychological impacts on this group higher either^29^. Our results indicated that more than half of the COVID-patients had psychological problems, accounting for >80,000 people worldwide at the moment, and this number would very likely soar in the following weeks. More strikingly, nearly one-fourth of the COVID-19 patients had at one point intended to conduct self-mutilation or suicide, and 28.3% had asked for psychological counseling. These findings address the importance of paying additional attention to these psychiatric morbidities when treating the physical problems in COIVD-19 patients.

When treating COVID-19 patients, we must not ignore the subsequent complications induced by psychiatric problems. Studies have shown that psychological stress can affect the immune system through neuroendocrine pathways^37,38^. IL-1β, TNF-α, IL-17, IL-6, and sIL-2R in the plasma and brain of patients with chronic depression are elevated, among which IL-1β is the main inflammatory cytokine of chronic stress response^39,40^. Anxiety can change the response of the sympathetic nervous system, which will result in the rise of systemic arterial pressure, increasing the heart rate^41,42^. An excessively fast heart rate will increase the left ventricular afterload and aggravate pulmonary edema, which will ultimately affect the respiratory functions of COVID-19 patients.

In addition, emotional and somatization symptoms not only affect the current rehabilitation process of the patients but also have certain impacts on the prognosis of the disease^43,44^. Psychological studies on Ebola patients have confirmed that psychological stress can persist during treatment and rehabilitation^45,46^. Anxiety, depression, and physical symptoms can also progress into chronic psychological problems. In the long run, these acute psychological problems would finally develop into chronic mental disorders, and even PTSD^47^. These mental disorders may be relieved by cognitive behavioral therapy, which was effective in reducing bad coping behaviors such as avoidance, confrontation, and self-blame by enhancing the patients’ ability to manage stress^48^.

Therefore, screening the concomitant psychological problems and providing mental health treatments for COVID-19 patients during their hospitalization is crucial, which could reduce the frequency of the patients revisiting doctors owing to emotional or somatization symptoms after discharge. Well-implemented, this additional screening may even reduce the wastes of medical resources and minimize medical disputes as well.

Several factors have possibly contributed to the psychiatric morbidities. Patients were facing a highly infectious novel virus that would lead to an imminent threat to their physical health. Compared with other disasters, the nature of this disease was totally unpredictable because COVID-19 was an unprecedented virus. Our results presented that the incidence of psychological problems was of high similarity between infected HCWs and non-HCWs, making it possible to draw an indication that all the people, with no exceptions, would fall into panic without the proper and sufficient preparations to combat this fatal, infectious disease. The extremely high mortality rate of the COVID-19 in the early stage may have been conducive to the high incidence of psychological problems in COVID-19 patients. Furthermore, the long incubation period and highly infectious nature of this disease make it prone to induce familial cluster infection. The fears of cross-infection to their family and friends may deteriorate their psychological well-being. Finally, increased quarantining was found to be significantly predictive of persistent depressive symptoms. Patients with isolation treatment would experience a longer period of not being able to have contact with their family members, as well as many other social supports.

Further analysis revealed that the depression and somatization symptoms of married patients were more severe when compared to unmarried patients. The primary means of SARS-CoV2 transmission is through respiratory droplets and direct contact as well as some unknown means^49^. The difference in the intensity level of the psychological symptoms of married patients may be related to the fear of transmitting to other family members. In addition, patients with positive nucleic acid tests have more severe depression. Patients who required oxygen inhalation developed more severe somatization symptoms. These several observations suggest that patients with severe illness are more prone to various psychological symptoms like somatization and depression. In conclusion, we should pay special attention to the mental health status of female married patients, patients who are nucleic acid-positive, and severe type individuals as we provide treatments to the COVID-19 patients.

The findings of this study have several limitations. One source of limitation is due to the exclusion of patients who were in critical conditions as a matter of ethics issues. This exclusion of partial population may have resulted in selective bias. Nevertheless, we found that the severity of psychological problems demonstrated a positive relationship with the severity of diseases for all the patients. In this case, the incidence of psychological problems could have been underestimated. A second limitation is due to the nature of this cross-sectional study: the basic mental health conditions of all patients could not be evaluated. Third, the mental health condition of COVID-19 patients could have also been affected by treatments, which is an aspect we have not investigated in the survey. Therefore, some of our findings need to be interpreted with a cautious mind.

## Conclusions

In summary, COVID-19 patients displayed a high incidence of anxiety, depression, and somatization. Early identification and intervention of the psychological problems in COVID-19 patients should be adopted to avoid extreme events such as self-mutilating or suicidal impulsivity of the patients, especially for those with low education levels and those females who have undergone divorce or bereavement.

## Supplementary information

Supplementary Materials

## Data Availability

All data generated or analyzed during this study are included in this published article.
