# Supplementary material for: Acute psychological impact on COVID-19 patients in Hubei: a multicenter observational study
Source: Transl Psychiatry. 2021 Feb 18;11:133. doi: 10.1038/s41398-021-01259-0 (PMC7890099; doi:10.1038/s41398-021-01259-0)
Supplement: Supplementary file 1 — Supplementary Materials [file 41398_2021_1259_MOESM1_ESM.docx]

**Supplementary materials**

**TABLE S1. Factors associated with the severity of suicide ideation in all COVID-19 patients.**

| Characteristics | Suicidal ideation | | *x*^2^***^b^*** | *p* |
| --- | --- | --- | --- | --- |
|  | No | Yes |  |  |
| **Gender** |  |  |  |  |
| Male | 135 | 28 | 5.233 | **0.022** |
| Female | 218 | 79 |  |  |
| **Age, year** |  |  |  |  |
| ≤17 | 63 | 16 | 0.547 | 0.761 |
| 18~44 | 170 | 52 |  |  |
| ≥45 | 120 | 39 |  |  |
| **Education level** |  |  |  |  |
| Senior high school or below | 103 | 58 | 22.607 | **<0.001** |
| Above Senior High school | 250 | 49 |  |  |
| **Marital status** |  |  |  |  |
| Unmarried | 85 | 15 | 13.61 | **0.001** |
| Married | 245 | 74 |  |  |
| Divorce / Bereavement | 23 | 18 |  |  |
| **Dwelling state** |  |  |  |  |
| Live alone | 63 | 21 | 0.174 | 0.676 |
| Live together | 290 | 86 |  |  |
| **Concomitant disease** |  |  |  |  |
| No | 253 | 72 | 0.76 | 0.383 |
| Yes | 100 | 35 |  |  |
| **Occupation** |  |  |  |  |
| Medical staff | 148 | 39 | 1.021 | 0.312 |
| Non-medical personnel | 205 | 68 |  |  |
| **Nucleic acid test** |  |  |  |  |
| Positive | 138 | 37 | 0.71 | 0.399 |
| Negative | 215 | 70 |  |  |
| **Fever** |  |  |  |  |
| Yes | 331 | 80 | 31.149 | **<0.001** |
| No | 22 | 27 |  |  |
| **Need oxygen inhalation** |  |  |  |  |
| No | 277 | 62 | 17.847 | **<0.001** |
| Yes | 76 | 45 |  |  |
| **Family members’ infection** |  |  |  |  |
| Confirmed infection | 110 | 24 | 13.852 | **0.001** |
| Suspected | 35 | 25 |  |  |
| No infection | 208 | 58 |  |  |
| **Psychological counseling** |  |  |  |  |
| No | 281 | 71 | 8.022 | **0.005** |
| Yes | 72 | 36 |  |  |

Note: ***b*** means chi square test. The severity of suicide ideation is related to gender (P=0.022), education level (P<0.001), marital status (P=0.001), Fever (P<0.001), Need oxygen inhalation (P<0.001), family infection (P=0.001) and Psychological counseling (P=0.005).

**TABLE S2. Factors associated with the severity of depression in all COVID-19 patients.**

| Characteristics | Severity of depression | | | | | *x*^2a^ | *p* |
| --- | --- | --- | --- | --- | --- | --- | --- |
|  | No | Mild | Moderate | Moderate severe | Severe |  |  |
| **Gender** |  |  |  |  |  |  |  |
| Male | 95 | 38 | 15 | 8 | 7 | 13.802 | **<0.001** |
| Female | 119 | 94 | 30 | 34 | 20 |  |  |
| **Age, year** |  |  |  |  |  |  |  |
| ≤17 | 44 | 20 | 5 | 9 | 1 | 3.542 | 0.17 |
| 18~44 | 99 | 62 | 24 | 23 | 14 |  |  |
| ≥45 | 71 | 50 | 16 | 10 | 12 |  |  |
| **Education level** |  |  |  |  |  |  |  |
| Senior high school or below | 63 | 51 | 18 | 17 | 12 | 5.518 | **0.019** |
| Above Senior High school | 151 | 81 | 27 | 25 | 15 |  |  |
| **Marital status** |  |  |  |  |  |  |  |
| Unmarried | 61 | 23 | 5 | 5 | 6 | 14.229 | **0.001** |
| Married | 139 | 100 | 35 | 29 | 16 |  |  |
| Divorce / Bereavement | 14 | 9 | 5 | 8 | 5 |  |  |
| **Dwelling state** |  |  |  |  |  |  |  |
| Live alone | 45 | 17 | 7 | 10 | 5 | 0.623 | 0.43 |
| Live together | 169 | 115 | 38 | 32 | 22 |  |  |
| **Concomitant disease** |  |  |  |  |  |  |  |
| No | 163 | 79 | 31 | 31 | 21 | 1.775 | 0.183 |
| Yes | 51 | 53 | 14 | 11 | 6 |  |  |
| **Occupation** |  |  |  |  |  |  |  |
| Medical staff | 90 | 50 | 16 | 20 | 11 | 0.065 | 0.799 |
| Non-medical personnel | 124 | 82 | 29 | 22 | 16 |  |  |
| **Nucleic acid test** |  |  |  |  |  |  |  |
| Positive | 78 | 56 | 18 | 13 | 10 | 0.032 | 0.859 |
| Negative | 136 | 76 | 27 | 29 | 17 |  |  |
| **Fever** |  |  |  |  |  |  |  |
| Yes | 200 | 120 | 42 | 28 | 21 | 14.589 | **<0.001** |
| No | 14 | 12 | 3 | 14 | 6 |  |  |
| **Need oxygen inhalation** |  |  |  |  |  |  |  |
| No | 184 | 85 | 33 | 22 | 15 | 31.253 | **<0.001** |
| Yes | 30 | 47 | 12 | 20 | 12 |  |  |
| **Family members’ infection** |  |  |  |  |  |  |  |
| Confirmed infection | 65 | 35 | 13 | 16 | 5 | 4.757 | 0.093 |
| Suspected | 18 | 27 | 2 | 8 | 5 |  |  |
| No infection | 131 | 70 | 30 | 18 | 17 |  |  |
| **Psychological counseling** |  |  |  |  |  |  |  |
| No | 179 | 98 | 28 | 29 | 18 | 12.942 | **<0.001** |
| Yes | 35 | 34 | 17 | 13 | 9 |  |  |

^a^ Means using rank sum test. The severity of depression is related to gender (P<0.001), education level (P=0.019), marriage (P=0.001), temperature (P<0.001), oxygen inhalation (P<0.001) and psychological counseling (P<0.001).

**TABLE S3. Factors associated with the severity of anxiety in all COVID-19 patients.**

| Characteristics | Anxiety severity | | | | *x*^2a^ | *p* |
| --- | --- | --- | --- | --- | --- | --- |
|  | No | Mild | Moderate | Severe |  |  |
| **Gender** |  |  |  |  |  |  |
| Male | 101 | 41 | 13 | 8 | 9.173 | **0.002** |
| Female | 146 | 81 | 37 | 33 |  |  |
| **Age, year** |  |  |  |  |  |  |
| ≤17 | 50 | 17 | 10 | 2 | 3.967 | 0.138 |
| 18~44 | 119 | 51 | 27 | 25 |  |  |
| ≥45 | 78 | 54 | 13 | 14 |  |  |
| **Education level** |  |  |  |  |  |  |
| Senior high school or below | 78 | 48 | 20 | 15 | 2.268 | 0.132 |
| Above Senior High school | 169 | 74 | 30 | 26 |  |  |
| **Marital status** |  |  |  |  |  |  |
| Unmarried | 74 | 12 | 6 | 8 | 18.371 | **<0.001** |
| Married | 156 | 100 | 37 | 26 |  |  |
| Divorce / Bereavement | 17 | 10 | 7 | 7 |  |  |
| **Dwelling state** |  |  |  |  |  |  |
| Live alone | 50 | 13 | 15 | 6 | 0.353 | 0.553 |
| Live together | 197 | 109 | 35 | 35 |  |  |
| **Concomitant disease** |  |  |  |  |  |  |
| No | 176 | 86 | 32 | 31 | 0.062 | 0.803 |
| Yes | 71 | 36 | 18 | 10 |  |  |
| **Occupation** |  |  |  |  |  |  |
| Medical staff | 103 | 46 | 21 | 17 | 0.087 | 0.767 |
| Non-medical personnel | 144 | 76 | 29 | 24 |  |  |
| **Nucleic acid test** |  |  |  |  |  |  |
| Positive | 95 | 50 | 18 | 12 | 0.357 | 0.55 |
| Negative | 152 | 72 | 32 | 29 |  |  |
| **Fever** |  |  |  |  |  |  |
| Yes | 231 | 109 | 40 | 31 | 14.535 | **<0.001** |
| No | 16 | 13 | 10 | 10 |  |  |
| **Need oxygen inhalation** |  |  |  |  |  |  |
| No | 205 | 81 | 28 | 25 | 24.874 | **<0.001** |
| Yes | 42 | 41 | 22 | 16 |  |  |
| **Family members’ infection** |  |  |  |  |  |  |
| Confirmed infection | 70 | 37 | 16 | 11 | 9.415 | **0.009** |
| Suspected | 20 | 26 | 7 | 7 |  |  |
| No infection | 157 | 59 | 27 | 23 |  |  |
| **Psychological counseling** |  |  |  |  |  |  |
| No | 210 | 87 | 27 | 28 | 23.195 | **<0.001** |
| Yes | 37 | 35 | 23 | 13 |  |  |

^a^ Means using rank sum test.

The severity of anxiety is related to gender (P=0.002), marriage (P<0.001), temperature (P<0.001), oxygen inhalation (P<0.001), family infection (P=0.009) and psychological counseling (P<0.001).

**TABLE S4. Factors associated with the severity of somatization symptoms in all COVID-19 patients.**

| Characteristics | Severity of somatization symptoms | | | | *x*^2a^ | *p* |
| --- | --- | --- | --- | --- | --- | --- |
|  | No | Mild | Moderate | Severe |  |  |
| **Gender** |  |  |  |  |  |  |
| Male | 77 | 51 | 25 | 10 | 31.24 | <0.001 |
| Female | 79 | 85 | 71 | 62 |  |  |
| **Age, year** |  |  |  |  |  |  |
| ≤17 | 33 | 15 | 15 | 16 | 5.357 | 0.069 |
| 18~44 | 63 | 69 | 53 | 37 |  |  |
| ≥45 | 60 | 52 | 28 | 19 |  |  |
| **Education level** |  |  |  |  |  |  |
| Senior high school or below | 52 | 54 | 24 | 31 | 0.106 | 0.744 |
| Above Senior High school | 104 | 82 | 72 | 41 |  |  |
| **Marital status** |  |  |  |  |  |  |
| Unmarried | 42 | 25 | 16 | 17 | 5.713 | 0.057 |
| Married | 103 | 103 | 70 | 43 |  |  |
| Divorce / Bereavement | 11 | 8 | 10 | 12 |  |  |
| **Dwelling state** |  |  |  |  |  |  |
| Live alone | 32 | 16 | 20 | 16 | 0.106 | 0.745 |
| Live together | 124 | 120 | 76 | 56 |  |  |
| **Concomitant disease** |  |  |  |  |  |  |
| No | 119 | 97 | 59 | 50 | 3.957 | 0.047 |
| Yes | 37 | 39 | 37 | 22 |  |  |
| **Occupation** |  |  |  |  |  |  |
| Medical staff | 62 | 49 | 44 | 32 | 0.978 | 0.323 |
| Non-medical personnel | 94 | 87 | 52 | 40 |  |  |
| **Nucleic acid test** |  |  |  |  |  |  |
| Positive | 52 | 59 | 42 | 22 | 0.225 | 0.636 |
| Negative | 104 | 77 | 54 | 50 |  |  |
| **Fever** |  |  |  |  |  |  |
| Yes | 146 | 127 | 89 | 49 | 18.754 | <0.001 |
| No | 10 | 9 | 7 | 23 |  |  |
| **Need oxygen inhalation** |  |  |  |  |  |  |
| No | 135 | 103 | 65 | 36 | 33.756 | <0.001 |
| Yes | 21 | 33 | 31 | 36 |  |  |
| **Family members’ infection** |  |  |  |  |  |  |
| Confirmed infection | 42 | 51 | 26 | 15 | 4.756 | 0.093 |
| Suspected | 17 | 15 | 9 | 19 |  |  |
| No infection | 97 | 70 | 61 | 38 |  |  |
| **Psychological counseling** |  |  |  |  |  |  |
| No | 131 | 108 | 72 | 41 | 16.303 | <0.001 |
| Yes | 25 | 28 | 24 | 31 |  |  |

^a^ Means using rank sum test.

The severity of somatization symptoms is related to gender (P<0.001), temperature (P<0.001), oxygen inhalation (P<0.001) and psychological counseling (P<0.001).

**TABLE S5. Factors associated with the severity of insomnia in all COVID-19 patients.**

| Characteristics | Insomnia severity | | | | *x*^2a^ | *p* |
| --- | --- | --- | --- | --- | --- | --- |
|  | No | Mild | Moderate | Severe |  |  |
| **Gender** |  |  |  |  |  |  |
| Male | 96 | 40 | 14 | 0 | 5.934 | **0.015** |
| Female | 140 | 66 | 40 | 11 |  |  |
| **Age, year** |  |  |  |  |  |  |
| ≤17 | 53 | 13 | 8 | 2 | 10.057 | **0.007** |
| 18~44 | 113 | 43 | 21 | 6 |  |  |
| ≥45 | 70 | 50 | 25 | 3 |  |  |
| **Education level** |  |  |  |  |  |  |
| Senior high school or below | 77 | 47 | 24 | 8 | 8.56 | **0.003** |
| Above Senior High school | 159 | 59 | 30 | 3 |  |  |
| **Marital status** |  |  |  |  |  |  |
| Unmarried | 63 | 8 | 13 | 3 | 10.733 | **0.005** |
| Married | 159 | 85 | 33 | 6 |  |  |
| Divorce / Bereavement | 14 | 13 | 8 | 2 |  |  |
| **Dwelling state** |  |  |  |  |  |  |
| Live alone | 41 | 17 | 10 | 3 | 0.061 | 0.805 |
| Live together | 195 | 89 | 44 | 8 |  |  |
| **Concomitant disease** |  |  |  |  |  |  |
| No | 173 | 70 | 32 | 9 | 3.204 | **0.073** |
| Yes | 63 | 36 | 22 | 2 |  |  |
| **Occupation** |  |  |  |  |  |  |
| Medical staff | 97 | 38 | 13 | 4 | 4.267 | **0.039** |
| Non-medical personnel | 139 | 68 | 41 | 7 |  |  |
| **Nucleic acid test** |  |  |  |  |  |  |
| Positive | 86 | 42 | 25 | 3 | 0.899 | 0.343 |
| Negative | 150 | 64 | 29 | 8 |  |  |
| **Fever** |  |  |  |  |  |  |
| Yes | 220 | 93 | 45 | 7 | 10.022 | **0.002** |
| No | 16 | 13 | 9 | 4 |  |  |
| **Need oxygen inhalation** |  |  |  |  |  |  |
| No | 192 | 77 | 32 | 6 | 13.684 | **<0.001** |
| Yes | 44 | 29 | 22 | 5 |  |  |
| **Family members’ infection** |  |  |  |  |  |  |
| Confirmed infection | 75 | 32 | 17 | 2 | 1.806 | 0.405 |
| Suspected | 26 | 21 | 6 | 2 |  |  |
| No infection | 135 | 53 | 31 | 7 |  |  |
| **Psychological counseling** |  |  |  |  |  |  |
| No | 208 | 86 | 33 | 10 | 14.194 | **<0.001** |
| Yes | 28 | 20 | 21 | 1 |  |  |

^a^ Means using rank sum test.

The severity of insomnia is related to gender (P=0.015), age (P=0.007), education level (P=0.003), marriage (P=0.005), occupation (P=0.039), temperature (P<0.002), oxygen inhalation (P<0.001) and psychological counseling (P<0.001).

**TABLE S6. Factors associated with stress response in all COVID-19 patients.**

| Characteristics | Stress response | | *x*^2^***^b^*** | *p* |
| --- | --- | --- | --- | --- |
|  | No | Yes |  |  |
| **Gender** |  |  |  |  |
| Male | 142 | 8 | 14.832 | **<0.001** |
| Female | 208 | 49 |  |  |
| **Age, year** |  |  |  |  |
| ≤17 | 64 | 12 | 0.375 | 0.829 |
| 18~44 | 157 | 26 |  |  |
| ≥45 | 129 | 19 |  |  |
| **Education level** |  |  |  |  |
| Senior high school or below | 138 | 18 | 1.278 | 0.258 |
| Above Senior High school | 212 | 39 |  |  |
| **Marital status** |  |  |  |  |
| Unmarried | 77 | 10 | 3.805 | 0.149 |
| Married | 245 | 38 |  |  |
| Divorce / Bereavement | 28 | 9 |  |  |
| **Dwelling state** |  |  |  |  |
| Live alone | 58 | 13 | 1.323 | 0.25 |
| Live together | 292 | 44 |  |  |
| **Concomitant disease** |  |  |  |  |
| No | 246 | 38 | 0.304 | 0.581 |
| Yes | 104 | 19 |  |  |
| **Occupation** |  |  |  |  |
| Medical staff | 133 | 19 | 0.456 | 0.499 |
| Non-medical personnel | 217 | 38 |  |  |
| **Nucleic acid test** |  |  |  |  |
| Positive | 132 | 24 | 0.4 | 0.527 |
| Negative | 218 | 33 |  |  |
| **Fever** |  |  |  |  |
| Yes | 314 | 51 | 0.003 | 0.956 |
| No | 36 | 6 |  |  |
| **Need oxygen inhalation** |  |  |  |  |
| No | 269 | 38 | 2.747 | 0.097 |
| Yes | 81 | 19 |  |  |
| **Family members’ infection** |  |  |  |  |
| Confirmed infection | 107 | 19 | 2.393 | 0.302 |
| Suspected | 51 | 4 |  |  |
| No infection | 192 | 34 |  |  |
| **Psychological counseling** |  |  |  |  |
| No | 303 | 34 | 24.948 | **<0.001** |
| Yes | 47 | 23 |  |  |

***^b^*** Means chi square test

The stress response is related to gender (P<0.001) and psychological counseling (P<0.001).
